# Hydroxysafflor yellow A for ischemic heart diseases: a systematic review and meta-analysis of animal experiments

**DOI:** 10.3389/fphar.2025.1510657

**Published:** 2025-04-09

**Authors:** Tianshi Mao, Kaixin Jiang, Yanting Pang, Yi Pan, Wenhao Jia, Qun Gao, Qian Lin

**Affiliations:** ^1^ Dongzhimen Hospital, Beijing University of Chinese Medicine, Beijing, China; ^2^ Department of Cardiology, Beijing University of Chinese Medicine Third Affiliated Hospital, Beijing, China; ^3^ Dongfang Hospital, Beijing University of Chinese Medicine, Beijing, China

**Keywords:** hydroxysafflor yellow A, myocardial ischemia, myocardial infarction, myocardial ischemia/reperfusion injury, meta-analysis

## Abstract

**Background:**

Hydroxysafflor yellow A (HSYA) possesses a variety of pharmacological activities which has been demonstrated to be effective against ischemic heart disease (IHD). This study aimed to comprehensively examine the efficacy and summarize the potential mechanisms of HSYA against IHD in animal models.

**Methods:**

We conducted electronic searches for preclinical studies on PubMed, Embase, Web of Science, Cochrane Library, CNKI, SinoMed, Wanfang, and Chinese VIP databases from inception to 31 January 2024. The CAMARADES checklist was chosen to assess the quality of evidence. STATA 14.0 software was utilized to analyze the data. The underlying mechanisms were categorized and summarized.

**Results:**

Twenty-eight studies involving 686 rodents were included and the mean score of methodology quality was 5.04 (range from 4 to 7). Meta-analysis observed that HSYA could decrease myocardial infarction size (SMD: −2.82, 95%CI: −3.56 to −2.08, *p* < 0.001) and reduce the levels of biomarkers of myocardial injury including cTnI (SMD: −3.82, 95%CI: −5.20 to −2.44, *p* < 0.001) and CK-MB (SMD: −2.74, 95%CI: −3.58 to −1.91, *p* < 0.001). HSYA displayed an improvement in cardiac function indicators including LVEF, LVSP, +dp/dt max and -dp/dt max. Furthermore, HSYA was able to reduce the levels of MDA, TNF-α and IL-6, while increasing SOD and NO levels. Mechanistically, the protective effect of HSYA in alleviating myocardial injury after ischemia may be associated with NLRP3 inflammasome, Bcl-2, Bax, caspase-3, eNOS proteins, and TLR/NF-κB, Nrf2/HO-1, JAK/STAT, PI3K/Akt, AMPK/mTOR, VEGFA pathways.

**Conclusion:**

This study demonstrates that HSYA exerts cardioprotective effects in decreasing infarct size, reducing myocardial enzymes and improving cardiac function, which may be mediated by anti-inflammatory, antioxidant, anti-apoptotic, regulation of autophagy, improvement of microcirculation and promotion of angiogenesis. However, the absence of safety assessment, lack of animal models of co-morbidities, and inconsistency between timing of administration and clinical practice are limitations of preclinical studies.

**Systematic Review Registration:**

clinicaltrials.gov, Identifier, CRD42023460790.

## 1 Introduction

Cardiovascular disease (CVD) remains a formidable threat to global public health. Despite considerable advancements in the treatment of CVD over the past 30 years, there is still an increasing trend in both incidence and mortality worldwide (Mensah et al., 2019). In 2020, the United States reported 127.9 million prevalent cases of CVD among adults, whereas there was a higher number of 330 million cases in China (Tsao et al., 2023; WCOTROCHADIC, 2023). Ischemic heart disease (IHD) is responsible for 49.2% of all CVD deaths and is also the leading cause of heart failure, accounting for 38.1%, which places a heavy burden on human health and medical care costs (Roth et al., 2020; Joseph et al., 2023). IHD comprises a range of myocardial ischemic diseases such as myocardial ischemia (IS), myocardial infarction (MI), and myocardial ischemia/reperfusion injury (MIRI) (Dong et al., 2023). In general, irreversible cardiomyocyte damage and death resulting from insufficient blood-oxygen supply are typical characteristics of IHD (Schirone et al., 2022). Therefore, several cardioprotective interventions aimed at ameliorating cardiac function by reducing cardiomyocyte injury and death have been implemented (Heusch, 2020).

As one of the oldest and most established medical systems in the world, traditional Chinese medicine (TCM) has been used and developed for more than 2,500 years. Currently, TCM plays an indispensable role in clinical treatment. According to the theory of TCM, IHD belongs to the category of “chest impediment disease”, and “blood stasis pattern” is the key pathogenesis. Based on pattern differentiation and treatment, “activating blood” and “resolving stasis” are the basic treatment principles of such disease. In the Compendium of Materia Medica (Ming Dynasty, ∼500 years ago), *Carthamus tinctorius* L. [Compositae; Carthami flos] is described as being able to “invigorate the blood circulation” (Cheng et al., 2024) and also becomes one of the frequently utilized botanical drugs for blood stasis disorders, suggesting its potential applications against IHD. The principal active product derived from safflower is called hydroxysafflor yellow A (HSYA), which serves as the designated marker metabolite utilized to measure the medicinal benefits of safflower in the 2020 edition of the *People’s Republic of China Pharmacopoeia* (Zhao et al., 2020). As reported, HSYA possesses a variety of pharmacological properties including antithrombotic, anti-inflammatory, antioxidant, anti-apoptotic, cardio-cerebral and even liver protection effects (Xue et al., 2021; Bai et al., 2020). For example, HSYA inhibits inflammation by regulating nod-like receptor protein-3 (NLRP3) inflammasome and suppressing nuclear factor kappa-B (NF-κB) signal pathway (Han et al., 2016; Ye et al., 2020). HSYA can also promote angiogenesis in the ischemic myocardium (Zou et al., 2018). Despite the facts that the therapeutic role and molecular mechanisms of HSYA for IHD have been partially elucidated, it is still difficult to transfer these findings from animals into humans. Certain scholars contend that a preclinical meta-analysis or systematic review is essential for improving the reproducibility and applicability of animal research (Spanagel, 2022). However, to the best of our knowledge, no review has systematically examined the effects of HSYA for IHD in animal models. Therefore, this systematic review aimed to investigate the therapeutic effects of HSYA for IHD and summarize the potential mechanisms, forming a chain of preclinical evidence and hoping to provide some ideas and research orientation for future preclinical research.

## 2 Materials and methods

### 2.1 Protocol

We have registered the protocol in the PROSPERO (CRD42023460790) and the status is “ongoing” now. Besides, the Preferred Reporting Items for Systematic Review and Meta-Analyses (PRISMA) 2020 statement and PRISMA-ATCM reporting guidelines was strictly followed in this study (Page et al., 2021; Zhao et al., 2022).

### 2.2 Search strategies

We did electronic searches across eight databases: PubMed, Embase, Web of Science, Cochrane Library, China National Knowledge Infrastructure, SinoMed, Wanfang, and Chinese VIP, from their inception up to 31 January 2024. The search entries were formulated as: (“hydroxysafflor yellow A” OR “HSYA”) AND (“myocardial ischemia” OR “myocardial infarction” OR “myocardial ischemia-reperfusion” OR “myocardial I/R”).

### 2.3 Inclusion criteria

The following prespecified inclusion criteria were stated: 1) The literature type was animal studies; 2) The research subjects were rats or mice, and the experimental animal models were IS, MI, or MIRI; 3) The intervention group received any dose of HSYA as monotherapy; 4) The control group was given a vehicle or no treatment; 5) The primary outcomes were myocardial infarct size (MIS), the serum level of cardiac troponin I (cTnI), creatine kinase myocardial band (CK-MB), CK, and lactic dehydrogenase (LDH). The secondary outcomes were left ventricular ejection fraction (LVEF), left ventricular systolic pressure (LVSP), left ventricular end dilated pressure (LVEDP), maximal left ventricular pressure rising rate (+dp/dt max) or maximal left ventricular pressure decreasing rate (-dp/dt max), the fibrosis of whole myocardium, the serum levels of tumor necrosis factor-α (TNF-α), interleukin 6 (IL-6), malondialdehyde (MDA), superoxide dismutase (SOD), 6-keto-prostaglandin F1α (6-keto-PGF1α), or nitric oxide (NO), the endothelial nitric oxide synthase (eNOS) activity in myocardium, the protein expression of B-cell lymphroma-2 (Bcl-2), Bcl-2-associated X protein (Bax), cleaved caspase-3 or light chain 3 (LC3), the mRNA level of nuclear factor erythroid 2-related factor 2 (Nrf2) or vascular endothelial growth factor A (VEGFA), and the apoptosis rate of cardiomyocytes.

### 2.4 Exclusion criteria

The following were preset criteria for exclusion: 1) Not *in vivo* studies: clinical trials, reviews, letter, meeting abstracts, *in vitro* studies; 2) Not rats or mice models with IS, MI and MIRI; 3) HSYA was not the only intervention; 4) The control group received any drugs treatment; 5) No one of the outcome indicators; 6) Studies with repeated publication of data; 7) Studies not available in full text.

### 2.5 Data extraction

This task was completed by two researchers independently, and the third researcher was responsible for resolving disagreements. The specifics of data extraction comprised: 1) General details: the first author’s name and publication year; 2) Animal information: species, gender, weight, and sample sizes; 3) Animal model: modeling methods and the anesthesia methods for model induction; 4) Intervention group: the dosage, administration method and time; 6) Control group: the vehicle name; 7) Outcome indicators.

We obtained data only from the highest dose group and final time point if the medication was administered at different doses or outcome indicators were recorded at multiple time nodes. The online tool WebPlotDigitizer was used to calculate the mean and standard deviation (SD) if the data was depicted graphically.

### 2.6 Risk of bias and quality assessment

To evaluate the risk of bias and methodological quality of enrolled studies, the grading scale of CAMARADES 10-item with minor modifications was employed (Macleod et al., 2004). The modifications are as follows: (F): use of anesthetic without significant intrinsic cardioprotective activity; (I): compliance with animal welfare regulations (including three or more of the following points: preoperative anaesthesia, postoperative analgesia, nutrition, disinfection, environment temperature, environment humidity, circadian rhythm, and euthanasia). Two individuals evaluated all enrolled literature independently.

### 2.7 Statistical analysis

Meta-analysis was conducted with the use of STATA 14.0 software. All outcome indicators belong to continuous variable type data and were shown as the standardized mean difference (SMD) along with a 95% confidence interval (95% CI). The statistical significance between the medication and control groups was set at *p* < 0.05. The effects model was selected depending on heterogeneity measures by Q test and *I* ^*2*^ statistics. A fixed effects model was applied to merge the effect size when the included studies had better homogeneity with *I* ^*2*^ ≤ 50% and *p* > 0.1. Otherwise, the random effects model was adopted (*I* ^*2*^ > 50% and *p* < 0.1). Meanwhile, we performed meta-regression and subgroup analyses to explore the probable reasons for heterogeneity. To assess the credibility of the pooled results, we further carried out the sensitivity analysis. When more than 10 studies were included, Egger’s test with *p* < 0.05 was denoted by a significant publication bias.

## 3 Results

### 3.1 Research selection

**FIGURE 1:**
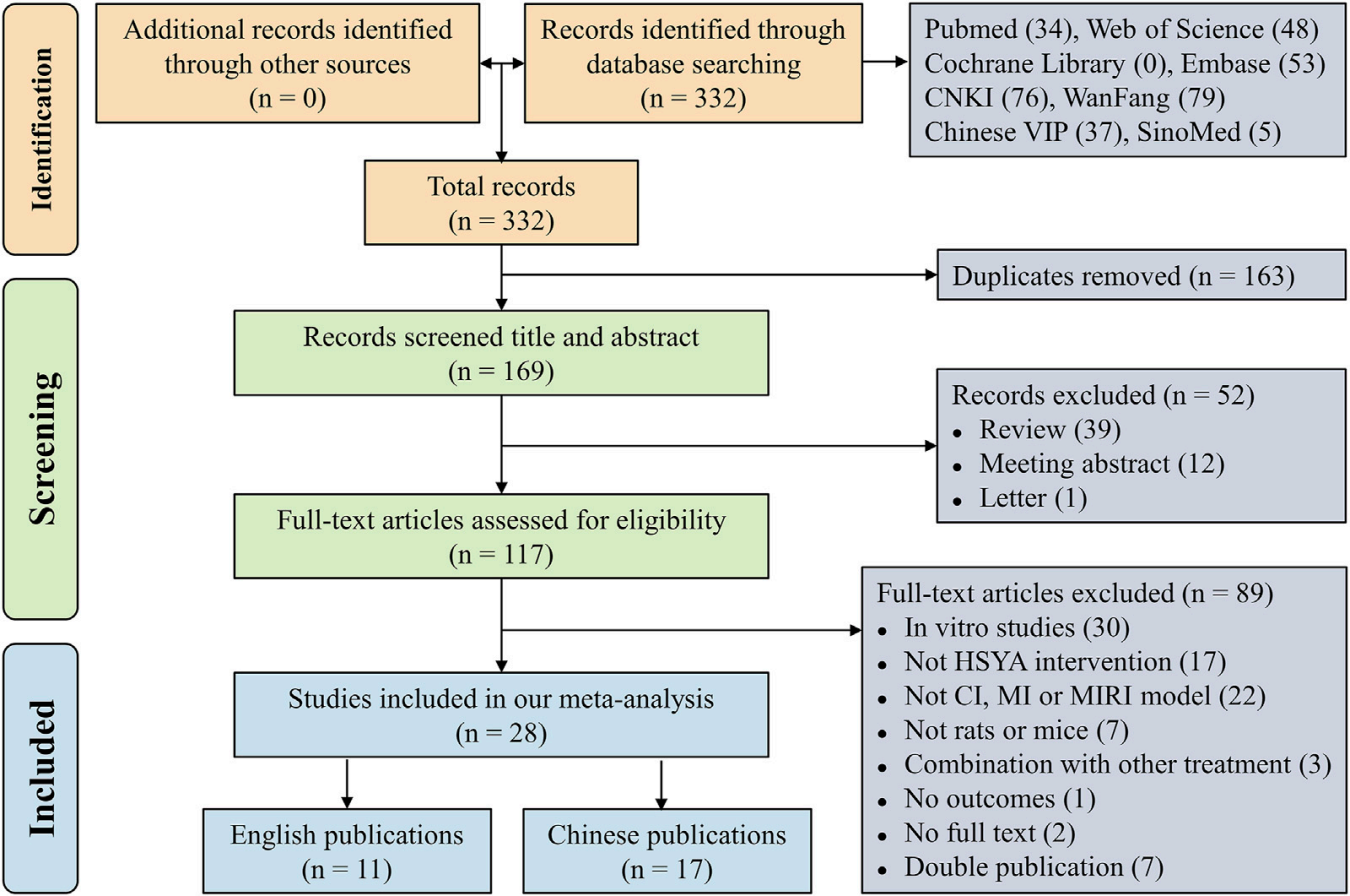
Flow diagram of the present systematic review and meta-analysis.

Initially, a total of 332 records were retrieved electronically. Then, 163 duplicate articles were excluded and there were 169 articles left. Next, 52 literature consisting of 39 reviews, 12 meeting abstracts and 1 letter were excluded after screening their titles and abstracts. Subsequently, 89 papers that met the exclusion criteria were excluded by skimming the whole text. Finally, our meta-analysis comprised 28 studies (Wang et al., 2007; Huang et al., 2009; Jin et al., 2009; Wang, 2009; Wang et al., 2009; Fu et al., 2011; Liu et al., 2011; Sun, 2012; Zhang et al., 2012; Guan et al., 2013; Dong et al., 2014; Xu et al., 2014; Chen, 2015; Hu et al., 2015; Liu et al., 2015; Zhou et al., 2015; Hu et al., 2016; Wei et al., 2016; Wei et al., 2017; Ni et al., 2018; Su et al., 2018; Zou et al., 2018; Zhou et al., 2019; Ye et al., 2020; Zhang and Zhang, 2020; He, 2022; Ge et al., 2023; Yu and Qu, 2023). Figure 1 displays the flowchart of literature selection.

### 3.2 Research quality

**FIGURE 2:**
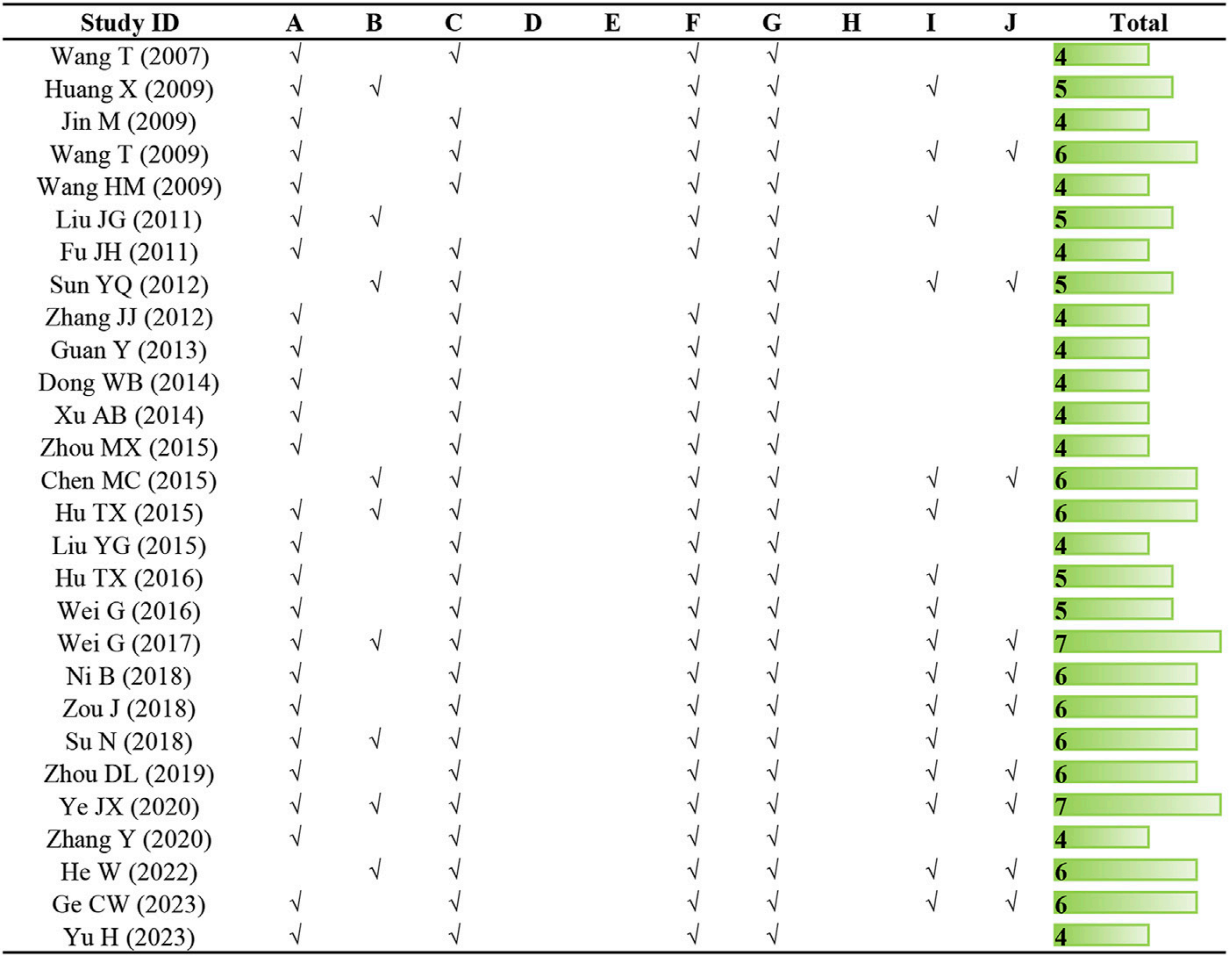
Risk of bias and quality assessment scores of included studies. Note: **(A)** peer review publication; **(B)** control of temperature; **(C)** random allocation to treatment or control; **(D)** blinded induction of model; **(E)** blinded assessment of outcome; **(F)** use of anesthetic without significant intrinsic cardioprotective activity; **(G)** appropriate animal model; **(H)** sample size calculation; **(I)** compliance with animal welfare regulations (including three or more of the following points: preoperative anesthesia, postoperative analgesia, nutrition, disinfection, environment temperature, environment humidity, circadian rhythm, and euthanasia); **(J)** statement of potential conflict of interests.

The quality scores of studies included in the meta-analysis ranged from 4 to 7, with an average of 5.04. Twenty-five studies underwent peer review. Only nine articles definitely mentioned temperature control, and two articles failed to describe the random allocation. Despite all studies employed appropriate animal models, blinded induction of model and blinded assessment of outcome were not mentioned in any of the studies. With the exception of one, all articles described the use of anesthetic without significant intrinsic cardioprotective activity. Each study provided information on the number of animals used, nonetheless, it is a pity that none of them calculated the sample size. Sixteen articles abided by the compliance with animal welfare regulations and ten literature declared potential conflict of interests. The methodological quality of each study is presented in Figure 2.

### 3.3 Research characteristics

Twenty-eight articles were included for meta-analysis, with 11 articles being English and the other 17 articles being Chinese publications. The detailed characteristics of the 28 articles are given in Table 1.

**TABLE 1:** Basic characteristics of included studies.

Study ID	Species (I/C)	Sex	Weight (g)	Model method	Anesthesia	Dosage (way)	Vehicle	Duration	Outcome
Wang et al. (2007)	Wister rats (10/10)	Male	250–300	LAD ligation	Urethanum	20 mg/kg iv	Saline	1d	(1), (3), (4), (15), (18)
Huang et al. (2009)	SD rats (7/7)	Male	200–240	LAD ligation	Sodium pentobarbital	77.3 mg/kg oral	Saline	0.5d	(1)
Jin et al. (2009)	Wister rats (8/8)	Male	200–250	ISO	Urethanum	240 mg/kg ip	Saline	3d	(20)
Wang et al. (2009)	SD rats (8/8)	Male	260–280	LAD ligation	Chloralose	8 mg/kg iv	NM	3d	(1), (3), (15), (16)
Wang, (2009)	SD rats (8/8)	Male	230–270	LAD ligation	Urethanum	20 mg/kg ip	Saline	4d	(15)
Liu et al. (2011)	SD rats (10/10)	Male	180–200	Ischemia 40 min, then reperfusion 120 min	Urethanum	17.87 g/kg iv	Saline	0.5d	(1), (3), (18)
Fu et al. (2011)	Wister rats (12/12)	Male	180–220	LAD ligation	Chloral hydrate	40 mg/kg ip	Saline	0.5d	(1), (3), (13), (14)
Sun, (2012)	SD rats (8/8)	Male	250–300	Ischemia 30 min, then reperfusion 24 h	NM	10 mg/kg iv	Saline	1d	(2), (4), (13), (14) (16), (20), (21), (22) (23), (24)
Zhang et al. (2012)	Wister rats (12/12)	Male, female	280–330	Ischemia 30 min, then reperfusion 120 min	Urethanum	16 mg/kg iv	Saline	0.5d	(1), (3), (18)
Guan et al. (2013)	SD rats (6/6)	NM	250–300	Ischemia 30 min, then reperfusion 3 h	Sodium pentobarbital	0.2 mg/kg iv	Saline	0.5d	(1), (3), (5), (7), (8), (9) (10), (11), (13), (14)
Dong et al. (2014)	SD rats (10/10)	Male, female	180–200	LAD ligation	Sodium pentobarbital	40 mg/kg iv	NM	3d	(1), (3), (4)
Xu et al. (2014)	SD rats (10/10)	Male, female	180–220	Ischemia 30 min, then reperfusion 3 h	Sodium pentobarbital	5 mg/kg iv	Saline	0.5d	(1), (3), (5), (7), (8), (9) (10), (11), (12)
Zhou et al. (2015)	Wister rats (31/31)	Male	80–120	LAD ligation	3.5% chloral hydrate	40 mg/kg ip	Saline	0.5d	(1)
Chen, (2015)	SD rats (6/6)	Male	230–270	ISO injection	10% chloral hydrate	100 mg/kg ig	Saline	14d	(20)
Hu et al. (2015)	SD rats (6/6)	Male	220–260	Ischemia 30 min, then reperfusion 180 min	Sodium pentobarbital	0.05 mg/kg iv	Saline	0.5d	(1), (3), (4), (5)
Liu et al. (2015)	SD rats (8/8)	Male	250–300	Ischemia 40 min, then reperfusion 4 h	Urethanum	16 mg/kg iv	Saline	0.5d	(1), (3), (4), (18)
Hu et al. (2016)	SD rats (10/10)	Male	230–270	Ischemia 30min, then reperfusion 180min	Sodium pentobarbital	70 mg/kg iv	NM	0.5d	(1), (3), (5), (13), (14)
Wei et al. (2016)	SD rats (12/12)	Male	220–260	LAD ligation	10% chloral hydrate	0.1 mg/kg iv	Saline	7d	(1), (6), (7), (8), (9) (10)
Wei et al. (2017)	C57BL/6 mice (40/40)	Male	25–30	LAD ligation	Sodium pentobarbital	60 mg/kg iv	Saline	28d	(6), (25)
Ni et al. (2018)	SD rats (10/10)	Male	275–300	LAD ligation	Isoflurane	5 mg/kg ip	NM	28d	(17)
Zou et al. (2018)	C57BL/6 mice (20/20)	Male	20–25	LAD ligation	Isoflurane	25 mg/kg ip	Saline	14d	(1), (7), (8), (9), (10) (19), (25)
Su et al. (2018)	C57BL/6 mice (10/10)	Male, female	18–22	LAD ligation	Isoflurane	10 mg/kg ip	DMSO	7d	(1), (11), (12), (21) (22)
Zhou et al. (2019)	SD rats (10/10)	Male	230–270	Ischemia 30min, then reperfusion 2 h	Sodium pentobarbital	5 mg/kg ip	Saline	0.5d	(1), (4), (5), (12) (13), (14), (20) (21), (22), (23)
Ye et al. (2020)	SD rats (15/15)	Male	260–300	Ischemia 30min, then reperfusion 24 h	Sodium pentobarbital	16 mg/kg iv	NM	0.5d	(1), (3), (4), (11), (20) (21), (22), (23), (24)
Zhang and Zhang (2020)	SD rats (10/10)	Male	180–200	Ischemia 30 min, then reperfusion 120 min	10% chloral hydrate	20 mg/kg ig	Saline	14d	(7), (8), (11), (12)
He, (2022)	C57BL/6 mice (16/16)	Male	18–22	LAD ligation	Isoflurane	10 mg/kg ip	DMSO	7d	(1), (3), (5), (11), (12) (15), (17)
Ge et al. (2023)	C57BL/6 mice (10/10)	Male	21–22	Ischemia 30min, then reperfusion 120min	Sodium pentobarbital	20 mg/kg ip	NM	14d	(1), (2), (4), (5), (19)
Yu and Qu (2023)	SD rats (20/20)	Male	NM	Ischemia 30min, then reperfusion 120min	Sodium pentobarbital	20 mg/kg ig	Saline	14d	(1), (2), (4), (5), (6) (13), (14), (21), (22)

*Note:* (1) MIS, myocardial infarct size; (2) CK, creatine kinase; (3) CK-MB, creatine kinase myocardial band; (4) LDH, lactic dehydrogenase; (5) cTnI, cardiac troponin I; (6) LVEF, left ventricular ejection fraction; (7) LVSP, left ventricular systolic pressure; (8) LVEDP, left ventricular end dilated pressure; (9) +dp/dt max; (10) -dp/dt max; (11) TNF-α, tumor necrosis factor-α; (12) IL-6, interleukin 6; (13) MDA, malondialdehyde; (14) SOD, superoxide dismutase; (15) NO, nitric oxide; (16) eNOS, endothelial nitric oxide synthase; (17) Nrf2, nuclear factor erythroid 2-related factor 2; (18) 6-keto-PGF1α, 6-keto-prostaglandin F1α; (19) fibrosis of myocardium; (20) apoptosis index; (21) Bax, Bcl-2-associated X protein; (22) Bcl-2, B-cell lymphroma-2; (23) cleaved caspase-3; (24) LC3II/LC3I, light chain 3II/light chain 3I; (25) VEGFA, vascular endothelial growth factor A; LAD: left anterior descending; NM: not mentioned; I: intervention group; C: control group; iv: intravenous; ip: intraperitoneal injection; ig: intragastric administration.

There were 686 included animals in total, of which 50.73% were Sprague-Dawley rats (348/686), 21.28% were Wister rats (146/686), and 27.99% were C57BL/6J mice (192/686). In terms of sex, the proportion of male and female animals were 92.13% (632/686) and 6.12% (42/686), respectively, while the left 1.75% (12/686) of animals were not mentioned sex. Regarding the methods of animal models establishment, 13 studies used only left anterior descending (LAD) ligation, 2 studies conducted by isoprenaline (ISO) injection, and 13 studies used the reperfusion after LAD ligation. To induce anesthesia, 11 studies used sodium pentobarbital, six studies used urethane, four studies used isoflurane, five studies utilized chloral hydrate, one study utilized chloralose, and the residual one study did not report the anesthetic. All studies were divided into three dose groups (low dose group: dosage ≤20 mg/kg/d, medium dose group: 20 mg/kg/d < dosage ≤40 mg/kg/d, high dose group: dosage >40 mg/kg/d) according to the dosage of HSYA, with 18 studies at low dose, four studies at medium dose, and six studies at high dose. The basic information about HSYA in each study are presented in Supplementary Table S1.

### 3.4 Primary outcome indicators

#### 3.4.1 Myocardial infarct size (MIS)

**FIGURE 3:**
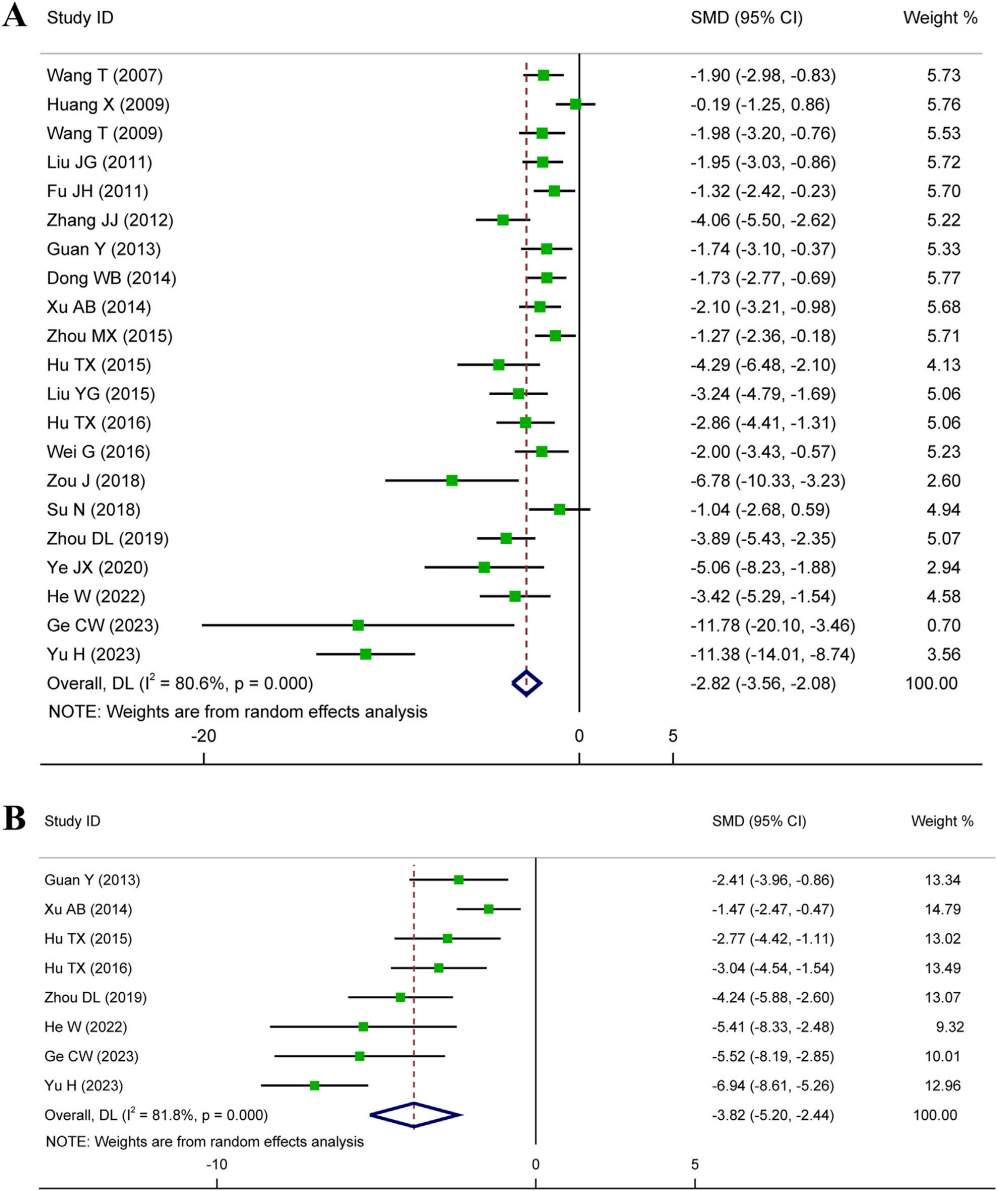
Forest plot presenting SMD and 95%CI for the effect of HSYA on MIS **(A)** and cTnI **(B)**.

**FIGURE 4:**
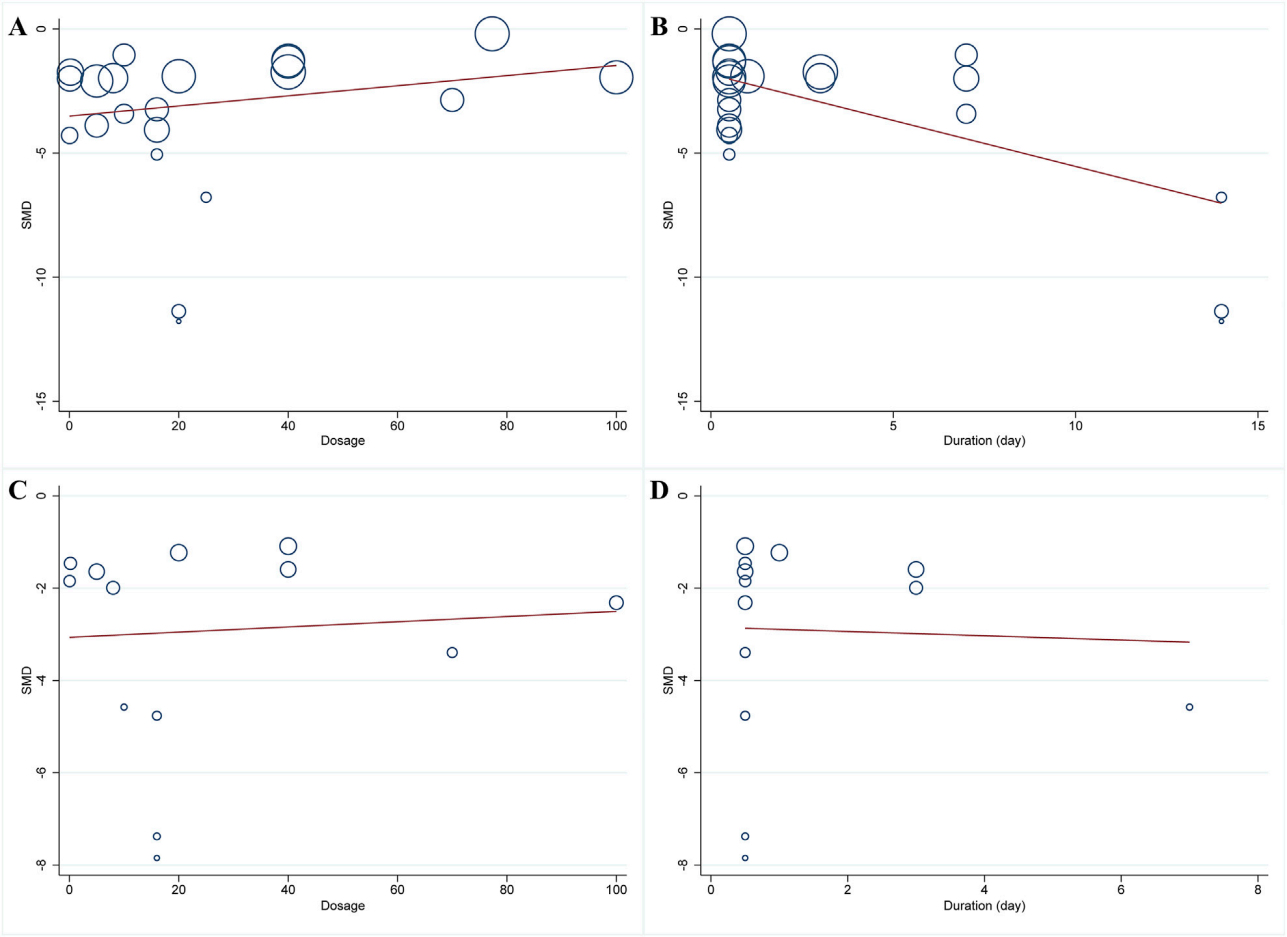
Meta-regression plots of MIS and CK-MB. **(A)** dosage and MIS; **(B)** treatment duration and MIS; **(C)** dosage and CK-MB; **(D)** treatment duration and CK-MB.

Meta-analysis of twenty-one studies proclaimed that HSYA had a significant effect on decreasing MIS in the animal models of IS, MI, and MIRI (n = 335, SMD: −2.82, 95% CI: −3.56 to −2.08, *p* < 0.001), with a high heterogeneity (*I* ^*2*^ = 80.6%, *p* < 0.001) (Figure 3A). To identify the potential reason of heterogeneity, the meta-regression analyses for dosage of HSYA and treatment duration were performed (Figures 4A, B). The results revealed that the treatment duration was the major cause of heterogeneity for MIS (*p* = 0.002) (Table 2).

**TABLE 2:** Meta-regression analysis.

Outcome	Factor	Coefficient (β)	Adj R^2^	95% CI	*p-*value
MIS	Dosage	0.0204	1.64%	−0.0200, 0.0608	0.304
	Duration	−0.3705	41.48%	−0.5923, −0.1488	**0.002**
CK-MB	Dosage	0.0056	−15.05%	−0.0414, 0.0527	0.797
	Duration	−0.0464	−12.36%	−0.0844, 0.7513	0.900

Abbreviations: MIS, myocardial infarct size; CK-MB, creatine kinase myocardial band.

#### 3.4.2 Myocardial enzymes

Myocardial enzymes are released after cardiomyocyte injury, and their levels are positively correlated with the degree of myocardial ischemia and injury.

##### 3.4.2.1 cTnI level

Eight articles detected the serum cTnI level, while an elevated heterogeneity was observed (*I* ^*2*^ = 81.8%, *p* < 0.001). The pooled results showed that compared with control, the medication group experienced a noticeable decrease in serum cTnI level (n = 142, SMD: −3.82, 95% CI: −5.20 to −2.44, *p* < 0.001) (Figure 3B).

##### 3.4.2.2 CK-MB level

**FIGURE 5:**
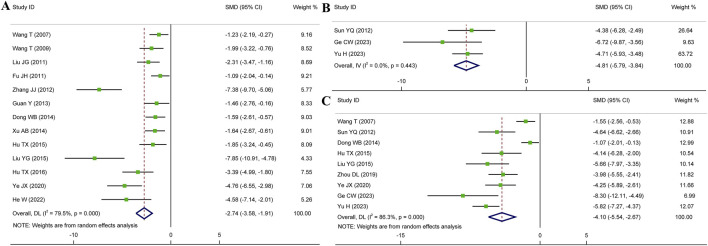
Forest plot presenting SMD and 95%CI for the effect of HSYA on the levels of CK-MB **(A)**, CK **(B)**, and LDH **(C)**.

A total of thirteen articles with an obvious heterogeneity (*I* ^*2*^ = 79.5%, *p* < 0.001) used the serum level of CK-MB as an outcome index. The meta-analysis results pointed to a significant reduction in CK-MB level with HSYA treatment (n = 226, SMD: −2.74, 95% CI: −3.58 to −1.91, *p* < 0.001) compared with the control group (Figure 5A). According to the results of meta-regression analysis shown in Figures 4C, D; Table 2, we found that the dosage of HSYA and duration were not the major factors of heterogeneity for CK-MB level.

##### 3.4.2.3 CK level

Overall three articles reported the serum level of CK, and a fixed effects model was employed due to low heterogeneity (*I* ^*2*^ = 0.0%, *p* = 0.443). Compared to the control group, HSYA exhibited a notable impact on decreasing serum CK level (n = 68, SMD: −4.81, 95% CI: −5.79 to −3.84, *p* < 0.001) (Figure 5B).

##### 3.4.2.4 LDH level

Nine articles were analyzed using a random effects model (*I* ^*2*^ = 86.3%, *p* < 0.001). We could observe that the serum levels of LDH were significantly decreased in HSYA group (n = 176, SMD: −4.10, 95% CI: −5.54 to −2.67, *p* < 0.001) (Figure 5C).

### 3.5 Secondary outcome indicators

#### 3.5.1 Cardiac function

**FIGURE 6:**
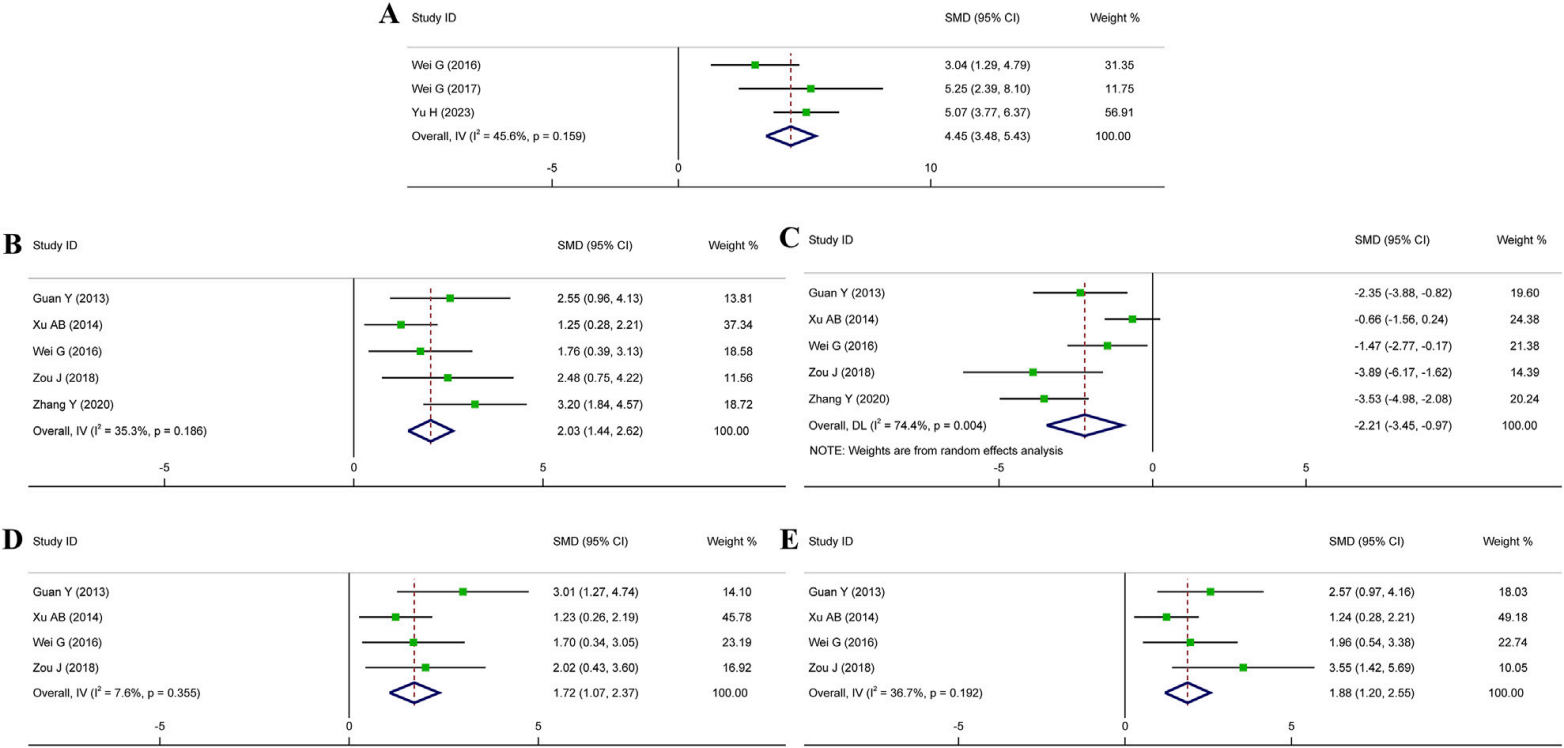
Forest plot presenting SMD and 95%CI for the effects of HSYA on cardiac function. **(A)** LVEF; **(B)** LVSP; **(C)** LVEDP; **(D)** +dp/dt max; **(E)** -dp/dt max.

To confirm the impact of HSYA therapy on cardiac function in MI, IS, and MIRI animal models, the related indices such as LVEF, LVSP, LVEDP, and ±dp/dt max were examined. Among these indicators, LVEF serves as an indicator of cardiac contractility. Meanwhile, LVSP, LVEDP, and ±dp/dt max represent the hemodynamic state and cardiac compliance of ventricle. Three research for LVEF, five for LVSP or LVEDP, and four studies for ± dp/dt max were brought into our meta-analysis. Figure 6 showed that HSYA treatment yielded remarkable improvements in LVEF (n = 62, SMD: 4.45, 95% CI: 3.48 to 5.43, *p* < 0.001; *I* ^*2*^ = 45.6%, *p* = 0.159), LVSP (n = 74, SMD: 2.03, 95% CI: 1.44 to 2.62, *p* < 0.001; *I* ^*2*^ = 35.3%, *p* = 0.186), LVEDP (n = 74, SMD: −2.21, 95% CI: −3.45 to −0.97, *p* < 0.001; *I* ^*2*^ = 74.4%, *p* = 0.004), +dp/dt max (n = 54, SMD: 1.72, 95% CI: 1.07 to 2.37, *p* < 0.001; *I* ^*2*^ = 7.6%, *p* = 0.355), and -dp/dt max (n = 54, SMD: 1.88, 95% CI: 1.20 to 2.55, *p* < 0.001; *I* ^*2*^ = 36.7%, *p* = 0.192). To summarize, HSYA treatment prominently improved cardiac dysfunction caused by myocardial injury. Specifically, HSYA enhanced myocardial contractility, consequently leading to an improvement in cardiac systolic function. It also improved cardiac hemodynamics and ventricular compliance, thereby contributing to the improvement of cardiac diastolic function.

#### 3.5.2 Inflammatory cytokines

Inflammation is the main factor of myocardial ischemic injury, and TNF-α and IL-6 are important mediators of inflammatory response. We analyzed data from six studies for TNF-α and five studies for IL-6. The pooled effects demonstrated that HSYA importantly reduced the levels of TNF-α (n = 80, SMD: −4.68, 95% CI: −6.40 to −2.95, *p* < 0.001; *I* ^*2*^ = 70.2%, *p* = 0.005) and IL-6 (n = 76, SMD: −3.98, 95% CI: −5.96 to −1.99, *p* < 0.001; *I* ^*2*^ = 81.9%, *p* < 0.001) in comparison to control group (Supplementary Figure S1). Our findings exhibited a significant therapeutic effect for anti-inflammation of HSYA in animal models.

#### 3.5.3 Oxidative stress indicators

To determine the relationship between HSYA treatment and oxidative stress levels, we used the following five indicators: the serum levels of MDA, SOD and NO, the protein eNOS activity, and the mRNA level of Nrf2 in myocardium. MDA and SOD are good indicators for evaluating oxidative stress, and Nrf2 is an important regulatory factor for antioxidant activity. The pooled estimates displayed that compared to control group, HSYA treatment could decrease MDA level (n = 124, SMD: −3.23, 95% CI: −4.39 to −2.06, *p* < 0.001; *I* ^*2*^ = 76.1%, *p* = 0.001), enhance SOD activity (n = 124, SMD: 2.73, 95% CI: 1.61 to 3.85, *p* < 0.001; *I* ^*2*^ = 78.4%, *p* < 0.001) and increase NO level (n = 62, SMD: 1.77, 95% CI: 1.15 to 2.39], *p* < 0.001; *I* ^*2*^ = 49.5%, *p* = 0.114). HSYA also showed a definite action on increasing eNOS activity (n = 32, SMD: 4.41, 95% CI: 1.27 to 7.55, *p* = 0.006; *I* ^*2*^ = 79.0%, *p* = 0.029) and Nrf2 mRNA level (n = 26, SMD: 8.01, 95% CI: 5.52 to 10.50, *p* < 0.001; *I* ^*2*^ = 0.0%, *p* = 0.734) (Supplementary Figure S2). Hence, it is confirmed that HSYA could alleviate oxidative stress levels in animal models of IHD.

#### 3.5.4 Platelet aggregation indicator

6-keto-PGF1α is a factor of platelet aggregation that reflects the blood circulation of myocardial. The reduced level gives rise to platelet aggregation and disorder of blood circulation, thereby exacerbating the extent of myocardial ischemia. A total of four studies reported the serum level of 6-keto-PGF1α as an outcome indicator and a random effects model was applied. According to the meta-analysis, HSYA increased serum level of 6-keto-PGF1α (n = 80, SMD: 2.51, 95% CI: 0.57 to 4.45, *p* = 0.011) compared to control and exhibited the inhibitory effect on platelet aggregation (Supplementary Figure S3A).

#### 3.5.5 Fibrosis of myocardium

Two studies applied Masson staining to detect the degree of myocardial injury and fibrosis. A fixed effects model was employed and the pooled results suggested that HYSA treatment alleviated myocardial injury and delayed the fibrosis progression (n = 16, SMD: −5.39, 95% CI: −7.77 to −3.00, *p* < 0.001) (Supplementary Figure S3B).

#### 3.5.6 Autophagy indicator

Two studies used the ratio of LC3II/LC3I as the outcome index and were analyzed by random effects model in this meta-analysis. The pooled results found no obviously statistical difference of HSYA on LC-3II/LC-3I ratio (n = 22, SMD: −15.02, 95% CI: −54.41 to 24.38, *p* = 0.455) in animal models of myocardial injury (Supplementary Figure S3C).

#### 3.5.7 Angiogenesis indicator

Two studies utilized VEGFA mRNA expression to evaluate the role of HSYA in angiogenic. The homogeneity between two studies was positive and the fixed effects model was utilized. The mRNA expression of VEGFA was significantly higher in HSYA group than in the control group (n = 20, SMD: 11.33, 95% CI: 7.30 to 15.36, *p* < 0.001), which suggests that HSYA is beneficial to the promotion of angiogenesis after myocardial injury in animal models (Supplementary Figure S3D).

#### 3.5.8 Apoptosis indicators

To investigate the correlation between HSYA treatment and cell apoptosis, we focused on some indicators of myocardial apoptosis. Five articles reported myocardial apoptosis rate by TUNEL assay, five articles provided data on Bax protein and Bcl-2 protein, as well as three articles on cleaved caspase-3. Results from eligible studies demonstrated that HSYA alleviated myocardial apoptosis (n = 70, SMD: −3.09, 95% CI: −4.55 to −1.64, *p* < 0.001; *I* ^*2*^ = 69.6%, *p* = 0.011), inhibited the activation of caspase-3 (n = 28, SMD: −9.94, 95% CI: −19.40 to −0.48, *p* = 0.039; *I* ^*2*^ = 90.1%, *p* < 0.001), reduced the Bax expression (n = 88, SMD: −5.28, 95% CI: −8.52 to −2.03, *p* = 0.001; *I* ^*2*^ = 91.0%, *p* < 0.001) and increased Bcl-2 expression (n = 88, SMD: 6.42, 95% CI: 4.46 to 8.39, *p* < 0.001; *I* ^*2*^ = 57.9%, *p* = 0.050) (Supplementary Figure S4). Taken together, HSYA could mitigate cardiomyocyte apoptosis *in vivo* of myocardial injury.

### 3.6 Subgroup analyses

Based on the potential factors (dosage, treatment duration, and stage of administration) that may contribute to heterogeneity, the subgroup analyses of MIS, cTnI, and CK-MB were conducted. In addition, considering that there were different methods of MIS calculation, subgroup analysis of MIS was also performed accordingly: area at infarction/area at risk (AAI/AAR), area at risk/left ventricle area (AAR/LVA), and area at risk/total myocardial area (AAR/TMA).

As presented in Table 3, the treatment duration was the major cause of heterogeneity for MIS, but the methods of MIS were not. It is consistent with the results of the meta-regression analysis. For CK-MB, the *p*-value of heterogeneity between groups was less than 0.01, indicating that the dosage of HSYA was the main source of heterogeneity. Similarly, the stage of administration may be the major source of heterogeneity for cTnI.

**TABLE 3:** Subgroup analysis.

Outcome	Number of studies	SMD	95% CI	*p*-value	Heterogeneity	
					I^2^	*p*
MIS						
Overall	21	−2.82	−3.56, −2.08	<0.001	80.6%	<0.001
Subgroup by (Duration), Heterogeneity between groups: *p* < 0.001						
≤7 days	18	−2.22	−2.73, −1.70	<0.001	60.9%	<0.001
>7 days	3	−9.65	−13.16, −6.13	<0.001	54.3%	0.112
Subgroup by (Methods of MIS), Heterogeneity between groups: *p* = 0.103						
AAI/AAR	5	−3.09	−4.01, −2.17	<0.001	34.5%	0.191
AAR/LVA	8	−2.21	−2.79, −1.63	<0.001	38.3%	0.124
AAR/TMA	8	−3.96	−5.90, −1.96	<0.001	91.1%	<0.001
CK-MB						
Overall	13	−2.74	−3.58, −1.91	<0.001	79.5%	<0.001
Subgroup by (Dosage), Heterogeneity between groups: *p* = 0.009						
≤20 mg/kg	9	−3.26	−4.50, −2.01	<0.001	83.9%	<0.001
20 ∼ ≤ 40 mg/kg	2	−2.71	−3.73, −1.69	<0.001	13.1%	0.283
>40 mg/kg	2	−1.32	−2.02, −0.63	<0.001	0.0%	0.478
cTnI						
Overall	8	−3.82	−5.20, −2.44	<0.001	81.8%	<0.001
Subgroup by (Stage of administration), Heterogeneity between groups: *p* < 0.001						
Therapeutic administration	4	−2.24	−3.01, −1.47	<0.001	20.0%	0.290
Prophylactic administration	4	−5.54	−6.91, −4.16	<0.001	40.9%	0.166

Abbreviations: MIS, myocardial infarct size; AAI, area at infarction; AAR, area at risk; LVA, left ventricle area; TMA, total myocardial area; CK-MB, creatine kinase myocardial band; cTnI, cardiac troponin I.

### 3.7 Publication bias and sensitivity analyses

For the primary outcome indicators MIS and CK-MB, the output results of publication bias using Egger’s test indicated the presence of publication bias (MIS: *t* = −4.85, *p* < 0.001; CK-MB: *t* = −7.85, *p* < 0.001) (Supplementary Figure S5).

**FIGURE 7:**
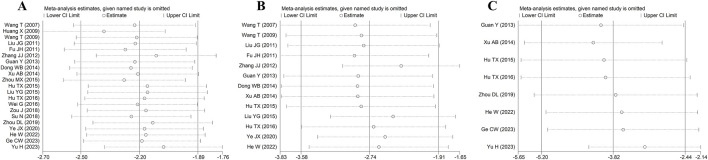
Sensitivity analysis of MIS **(A)**, CK-MB **(B)**, and cTnI **(C)**.

Owing to the existence of heterogeneity and publication bias, we completed the sensitivity analyses using the leave-one-out method to assess the robustness of MIS, CK-MB, and cTnI. The results of sensitivity analyses showed that the overall effect size was not significantly changed, which implied that our findings of this study were relatively stable and reliable (Figure 7).

## 4 Discussion

### 4.1 Summary of evidence

Up to now, this is the first preclinical meta-analysis and systemic review to examine the effect of IHD following HSYA treatment *in vivo*. A previous study (Zhao et al., 2020) systematically summarized the basic data for related research of HSYA, including pharmacological effects and molecular mechanism. But it focused more on reporting the methods of acquisition, extraction and detection, as well as pharmacokinetics. Moreover, regarding the cardioprotective effects of HSYA, it only summarized oxidative stress and inflammation related signaling pathways. In our review, we provided evidence about the efficacy of HSYA against IHD through meta-analysis and updated a more comprehensive review of pharmacological mechanisms.

**FIGURE 8:**
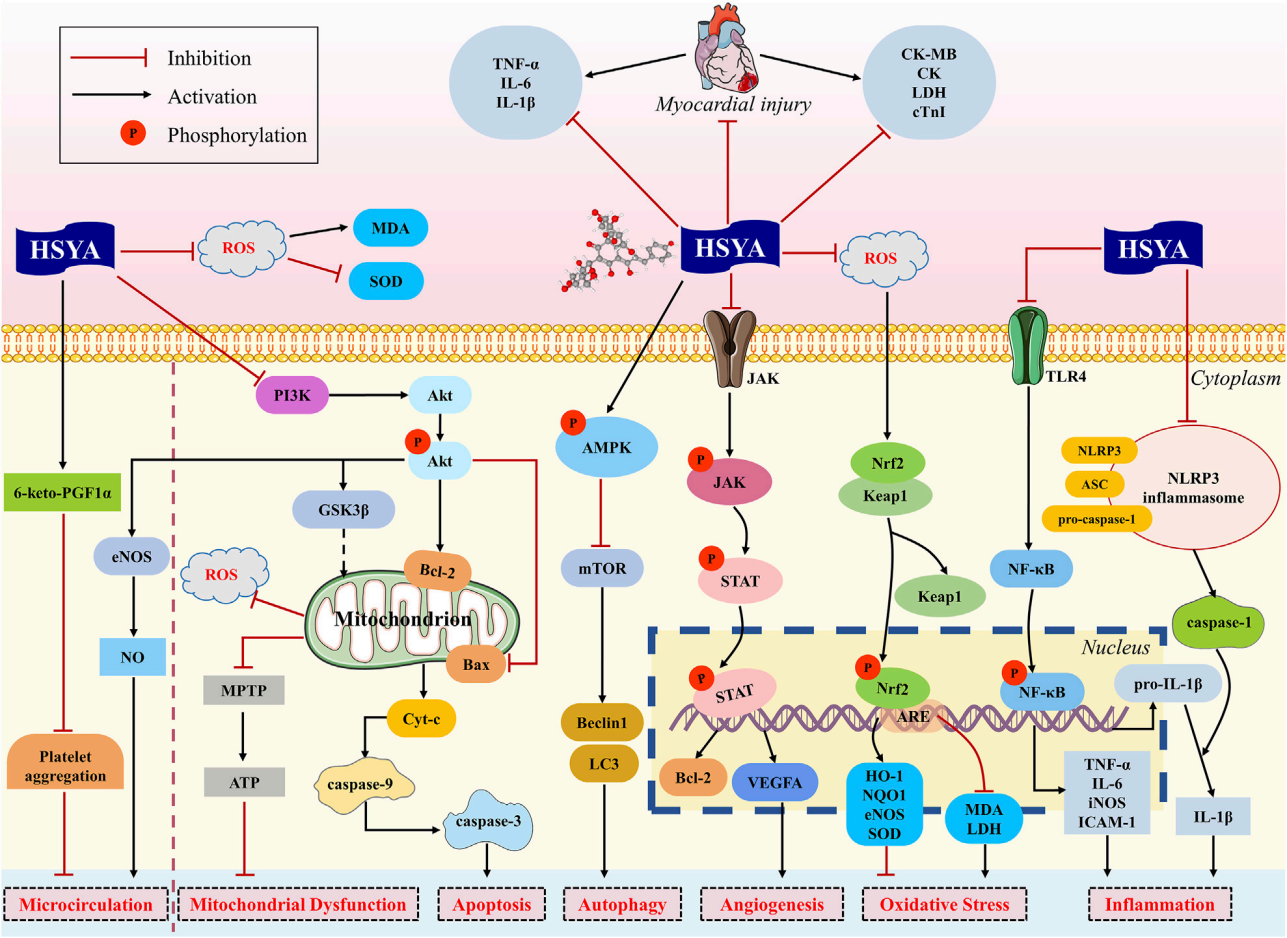
Potential cardioprotective mechanisms of HSYA against myocardial ischemia.

Our findings found that HSYA is devoted to reducing MIS, serum myocardial enzymes and troponin, enhancing cardiac function, decreasing serum inflammatory cytokines, and ameliorating myocardial apoptosis and fibrosis. In brief, HSYA exerts potential cardioprotective action against myocardial ischemic injury mainly through anti-inflammatory, antioxidant, anti-apoptotic, regulation of autophagy, improvement of microcirculation and mitochondrial dysfunction, and promotion of angiogenesis (Figure 8).

### 4.2 Molecular mechanisms of HSYA for myocardial ischemia

#### 4.2.1 Effects on inflammation

During the process of myocardial ischemia, inflammatory cells infiltrate the infarct region and promote the release of inflammatory mediators. The inflammatory responses localized at the myocardium are triggered, which is considered to be the primary factor responsible for myocardial damage and aggravated cardiac dysfunction (Ong et al., 2018).

The toll-like receptor 4 (TLR4)/NF-κB signal pathway contributes an important part in inflammation. TLR4, as a pattern recognition receptor located on the cell membrane, participates in the inflammatory response actively (Wei et al., 2023). In case of myocardial injury, TLR4 activation triggers the downstream signaling p-NF-κB, bringing about the secretion of proinflammatory cytokines such as TNF-α, IL-6, IL-1β, and inducible nitric oxide synthase (iNOS) (Moghimpour Bijani et al., 2012). This inflammatory cascade worsens myocardial damage and is likely to induce an enlargement of infarct size. It was reported that HSYA attenuated TLR4 overexpression and NF-κB activation, contributing to the downregulation of TNF-α, IL-6 and IL-1β, and the upregulation of anti-inflammatory cytokine IL-10, which in turn inhibits the inflammatory response of cardiomyocytes (Guan et al., 2013; Han et al., 2016).

Moreover, recent preclinical studies of MI and MIRI have identified that NLRP3 inflammasome is also closely connected to heart inflammation, infarct size, and cardiac function (Toldo et al., 2016). NLRP3 inflammasome is composed of three components: NLRP3 protein, apoptosis-associated speck-like (ASC) protein, and pro-caspase-1 (Abderrazak et al., 2015). Ischemia can trigger NLRP3 activation, which contributes to the production and release of IL-1β (Xu et al., 2020; Dai et al., 2023). HSYA effectively suppressed NLRP3 inflammasome activation in rats with MIRI, resulting in decreased IL-1β release and infarction size (Ye et al., 2020).

#### 4.2.2 Effects on oxidative stress

Excessive production of reactive oxygen species (ROS) is the main pathophysiological factor implicated in the progression of MI and MIRI (Peoples et al., 2019). During myocardial ischemia, the amount of ROS increases and antioxidant systems are impaired. The redox transcription factor Nrf2 is essential to the regulation of cellular redox homeostasis, thereby exhibiting the effect of antioxidant stress. Keap1 is a vital element for the regulation of Nrf2. Under normal conditions, Nrf2 is bound to Keap1, which promotes the proteasomal degradation of Nrf2 by way of ubiquitination, keeping Nrf2 maintain a low level in the cytoplasm. But in oxidative stress conditions, Nrf2 dissociates from its complex pair and translocates to the nucleus. Once Nrf2 is transported to the nucleus, it always binds to the sequence known as the antioxidant response element (ARE), boosting the transcription of Nrf2-regulated genes (Loboda et al., 2016; Geertsema et al., 2023). Two downstream targets of Nrf2, heme oxygenase-1 (HO-1) and NAD(P)H-quinone oxidoreductase 1 (NQO1) are believed to possess a protective effect on oxidative stress. HSYA has been shown to reduce oxidative stress damage in mice which is achieved by inhibiting the level of Keap1 mRNA and enhancing the level of Nrf2 and HO-1 (He, 2022). As well, HSYA also exerts antioxidant and anti-hypertrophic effects in rats with MI by activating Nrf2/HO-1 signaling pathway (Ni et al., 2018).

#### 4.2.3 Effects on apoptosis and mitochondrial dysfunction

Apoptosis is recognized to involve in the progression of myocardial ischemia (Singh et al., 2019). Cardiomyocyte apoptosis begins shortly following the onset of MI and appears to be enhanced apparently during reperfusion, leading to the stasis of myocardium, the enlargement of infarct area, and the insufficiency of cardiac function (Zhao et al., 2003).

The overproduction of ROS is the major apoptotic stimulus signal. Subsequently, the apoptotic cascade is initiated, which involves the loss of mitochondrial membrane potential, the opening of mitochondrial permeability transition pore (MPTP), and the release of cytochrome-C (Cyt-C) (Heusch, 2020). Cyt-C can combine with apoptotic protease activating factor-1 (Apaf-1), causing the sequential activation of caspase-3, which is subsequently processed into cleaved caspase-3. The activated and cleaved caspase-3 is regarded as an indicator of the intensity of apoptosis (Sahoo et al., 2023). Bcl-2 and Bax are crucial mitochondrial membrane proteins and serve as the integral regulators of apoptosis, which are responsible for maintaining mitochondrial membrane integrity and the balance of apoptosis (Czabotar and Garcia-Saez, 2023; Senichkin et al., 2020). Bcl-2 can block Cyt-C release and regulate MPTP opening to inhibit apoptosis, whereas Bax performs the opposite effect. HSYA was found to reduce Cyt-C release, increase the expression level of Bcl-2, and decrease the expression of Bax and caspase-3 *in vivo*, thereby inhibiting cardiomyocyte apoptosis (Sun, 2012; Zhou et al., 2019; Ye et al., 2020).

Janus kinase (JAK)/signal transducers and activators of transcription (STAT) signal pathway is essential for cell apoptosis and ischemia injury (Pang et al., 2023). JAK2 could be activated by ROS and then STAT1 is phosphorylated by JAK2. Previous research identified that STAT1 could modulate MPTP opening and enhance the expression of pro-apoptotic genes such as p53, Fas, and Fas ligand (FasL) (Krämer et al., 2006). HSYA decreased JAK2 and STAT1 activity, inhibited the expression of Bax, Fas, and FasL, improved antioxidant capacity, and reduced apoptosis in IR-induced models. It was suggested that HSYA is effective in ameliorating myocardial injury by inhibiting apoptosis, which is partially mediated by JAK2/STAT1 pathway (Zhou et al., 2019).

Mitochondrial dysfunction is among the pathophysiological mechanisms involved in MIRI. During acute ischemia, the overproduction of ROS causes an overload of mitochondrial Ca^2+^, which promotes the opening of MPTP, leading to impaired adenosine triphosphate (ATP) synthesis and mitochondrial swelling (Halestrap et al., 2004). Therefore, the formation and opening of MPTP are the major causes of mitochondrial dysfunction and cardiomyocyte apoptosis (Deng and Zhou, 2023). HSYA has been shown to prevent myocardial injury through inhibiting the opening of MPTP in isolated rat hearts (Liu et al., 2008). Phosphatidylinositol 3-kinase (PI3K)/protein kinase B (Akt)/glycogen synthase kinase three beta (GSK3β) signaling pathway has been claimed to benefit mitochondrial and myocardial damage (Zhang et al., 2018). GSK3β, as the key downstream molecule of PI3K/Akt pathway, has been proven to regulate MPTP opening. It has been reported that HSYA increases levels of p-Akt and GSK3β in H/R-induced H9c2 cells, which inhibits cardiomyocyte apoptosis, prevents MPTP opening, and restores mitochondrial ATP synthesis (Min and Wei, 2017).

#### 4.2.4 Effects on microcirculation

During the progression of MI and MIRI, partly coronary microvascular cells also may experience irreversible damage except for cardiomyocyte death, leading to microcirculation dysfunction (Heusch, 2019). Platelet aggregation is a major factor in microcirculation disturbance. 6-keto-PGF1α is a derivative of prostaglandin I_2_ (PGI_2_), which is an important active substance that inhibits platelet aggregation. Studies found that 6-keto-PGF1α decreased in rats with MI and MIRI, while HSYA could increase the level of 6-keto-PGF1α (Wang et al., 2007; Liu et al., 2011; Zhang et al., 2012). Additionally, as a natural inorganic compound to protect against platelet aggregation, NO is produced in vascular endothelium by eNOS (Lundberg and Weitzberg, 2022). It was discovered that HSYA could attenuate the decrease of eNOS activity caused by acute myocardial ischemia and consequently enhance NO content in MI rats (Wang et al., 2009). This cardioprotective effect is claimed to be relevant to the promotion of the PI3K/Akt/eNOS pathway after HSYA treatment in MIRI rats (Sun, 2012).

#### 4.2.5 Other effects

Angiogenesis is an essential process in the recovery of blood flow in ischemic myocardial tissue. It helps to establish collateral circulation and further promotes myocardial regeneration (Ware and Simons, 1997). Currently, plenty of evidence has reported the proangiogenic effect of HSYA by using various ischemic models. In MI mice, HSYA has performance in promoting myocardial neovascularization and recovering heart function, which might be attributed to the activation of HO-1/VEGF/SDF-1 cascade pathway (Wei et al., 2017). Furthermore, HSYA could upregulate VEGFA expression to promote angiogenesis in ischemic myocardium, thereby ameliorating ischemia-induced cardiac dysfunction and myocardial injury (Zou et al., 2018).

Autophagy is a major regulator of cardiac homeostasis in response to various stressful conditions, thereby reducing myocardial injury and protecting cardiac function (Sciarretta et al., 2018). The mammalian target of rapamycin (mTOR) is a well-known inhibitor of autophagy, whereas AMP-activated protein kinase (AMPK), the upstream of mTOR, is the positive regulator. It was found that HSYA could increase p-AMPK level and decrease mTOR level to stimulate cardiomyocyte autophagy in MIRI rats. Moreover, the autophagy-related proteins were observed, with Beclin one and LC3II showing an increasing trend while LC3I decreased in rats receiving HSYA (Ye et al., 2020). However, the specific mechanisms of HSYA regulating autophagy are not well understood and require further experiments.

### 4.3 Limitations

Firstly, nearly 50% of the studies had a quality score of less than five points, indicating an overall low quality of all included studies. Low methodological quality indeed affects the credibility of the results.

Secondly, most outcome measures in this study exhibited high heterogeneity, which could be attributed to a few factors such as animal species, drug dosage, modeling methods, and measurement errors. However, we further performed meta-regression, subgroup analysis, and sensitivity analysis, and the results validated the effectiveness and reliability of HSYA for treating myocardial ischemia.

Thirdly, it was found a significant publication bias for MIS and CK-MB. Those studies not with positive results are rarely published, especially animal experiments. Thus, the efficacy of HSYA may be overestimated. We controlled for publication bias by using as many database sources as possible, but we could not collect all the relevant literature.

Lastly, none of the preclinical studies reported the assessment of safety, which makes us fail to provide evidence of safety through meta-analysis.

### 4.4 Future prospects

Despite HSYA has been investigated the diverse pharmacological activities, it is well known for being a pan-assay interference compound (PAINS) if tested *in vitro*. PAINS are molecules that have been observed to show activity in multiple types of assays by interfering with the assay readout rather than through specific compound/target interactions, which are often viewed as highly promiscuous players that produce false positive results (Magalhães et al., 2021).

PAINS always exhibit some known typical behaviors: redox reactivity, aggregation, membrane disruption, and fluorescence interference (Nelson et al., 2017). *In vitro*, PAINS may interfere with oxidative stress-related detection by scavenging free radicals (redox reactivity); PAINS could lead to false results through nonspecific bind to antibodies in some biochemical assays (aggregation); color or fluorescence properties of PAINS may produce an effect on fluorescence signal (fluorescence interference). *In vitro* experiments are usually performed under simplified conditions that lack the complexities of environment *in vivo*, such as inability to be reduced by hepatic metabolic enzymes that may interfere with the assay in prototype form. Additionally, the concentrations of PAINS were used ranging from μM to mM levels, which significantly exceed the physiological concentrations observed *in vivo*, thereby greatly increasing the probability of nonspecific reactions (Bolz et al., 2021). It is obvious that the *in vitro* interference properties of HSYA may offer many traps of investigations.

However, PAINS have relatively little interference with the *in vivo* experiments. Firstly, HSYA undergoes hydroxylation and glucuronidation under the action of hepatic enzymes (Zhao et al., 2020). The metabolites decrease the nonspecific reactivity on oxidative stress detection. Furthermore, *in vivo* experiments rely on a comprehensive phenotype to assess efficacy of PAINS, rather than a single biochemical test. For instance, MIS, the primary outcome of this meta-analysis, was assessed by TTC staining and was not directly interfere by redox properties of PAINS. Cardiac function parameters (LVEF, +dp/dt max, etc.) were also not affected by the activity of single or multiple targets. Taken together, the integration of evidence from animal research may further enhance confidence in our results. But this does not mean that PAINS will not have interference effects at all *in vivo* experiments, and future research need to further ensure the reliability of results through conducting pharmacokinetic studies and looking for biophysical orthogonal methods for support of target engagement (e.g., surface plasmon resonance, cellular thermal shift assay) (Dahlin et al., 2015).

Moreover, the further preclinical research should adopt more rigorous experimental design such as blinded implementation and sample size calculation. Safety assessment should also be one of the concerns for each research. In addition, how to resolve the inconsistency between the timing of administration and clinical practice remains a key issue for future research.

## 5 Conclusion

In summary, our review findings indicate that HSYA exerts cardioprotective benefits in decreasing myocardial infarct size, reducing myocardial enzymes, and improving cardiac function in animal models of IS, MI, and MIRI. Specifically, the potential effects of HSYA in alleviating myocardial injury after ischemia mainly include attenuating inflammation, oxidative stress, cardiac myocyte apoptosis, and fibrosis of myocardium, improving microcirculation, and promoting angiogenesis. The underlying mechanisms may be associated with NLRP3 inflammasome, Bcl-2, Bax, caspase-3, eNOS proteins, and TLR/NF-κB, Nrf2/HO-1, JAK/STAT, PI3K/Akt, AMPK/mTOR, VEGFA pathways. However, none of included studies reported the safety endpoint and used animals with co-morbidities such as hyperlipidemia and hypertension, which is the typical situation in human for IHD. Moreover, the timing of administration of HSYA does not align with clinical practice. Safety assessment, standardized experimental design and comorbid animal models may strengthen the preclinical evidence base. Therefore, stronger evidence is required to better convert these finding to clinical practice.

## Data Availability

The original contributions presented in the study are included in the article/Supplementary Material, further inquiries can be directed to the corresponding authors.
