# Supplementary material for: Hydroxysafflor yellow A for ischemic heart diseases: a systematic review and meta-analysis of animal experiments
Source: Front Pharmacol. 2025 Apr 9;16:1510657. doi: 10.3389/fphar.2025.1510657 (PMC12014549; doi:10.3389/fphar.2025.1510657)
Supplement: Supplementary file 1 [file DataSheet1.docx]

**Supplementary material**

**Figure S1.** Forest plot presenting SMD and 95%CI for the effects of HSYA on inflammatory cytokines. (A) TNF-α; (B) IL-6.

**Figure S2.** Forest plot presenting SMD and 95%CI for the effects of HSYA on oxidative stress. (A) MDA level; (B) SOD level; (C) NO level; (D) eNOS activity; (E) Nrf2 mRNA level.

**Figure S3.** Forest plot presenting SMD and 95%CI for the effects of HSYA on 6-keto-PGF1α level (A), myocardium fibrosis (B), LC-3II/LC-3I ratio (C), and VEGFA mRNA level (D).

**Figure S4.** Forest plot presenting SMD and 95%CI for the effects of HSYA on apoptosis. (A) apoptosis index, (B) cleaved caspase-3 protein; (C) Bax protein; (D) Bcl-2 protein.

**Figure S5.** Egger’s test of MIS (A) and CK-MB (B).

**Table S1.** Detailed information of HSYA in each study.

**
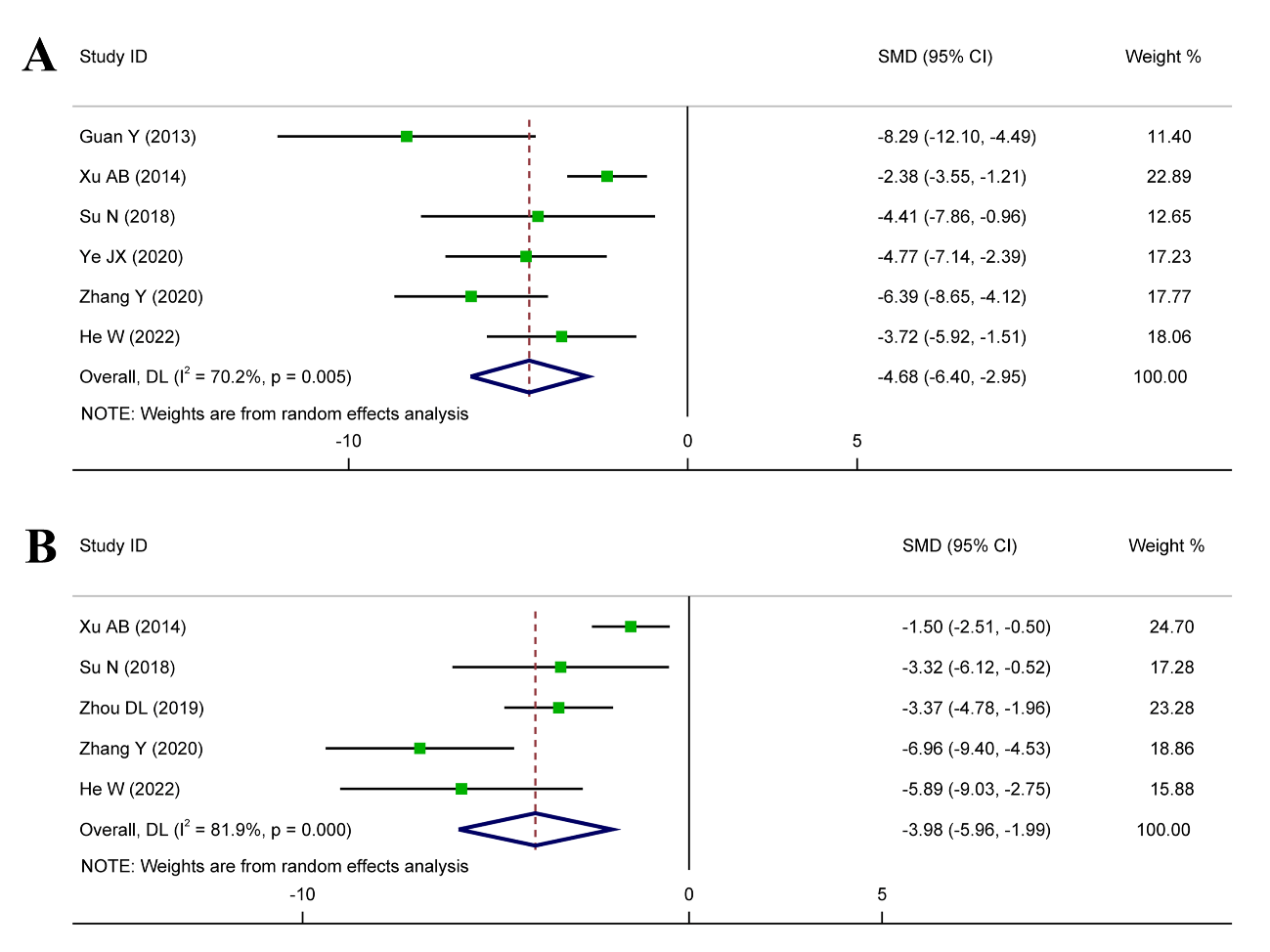
**

**Figure S1.** Forest plot presenting SMD and 95%CI for the effects of HSYA on inflammatory cytokines. (A) TNF-α; (B) IL-6.

**
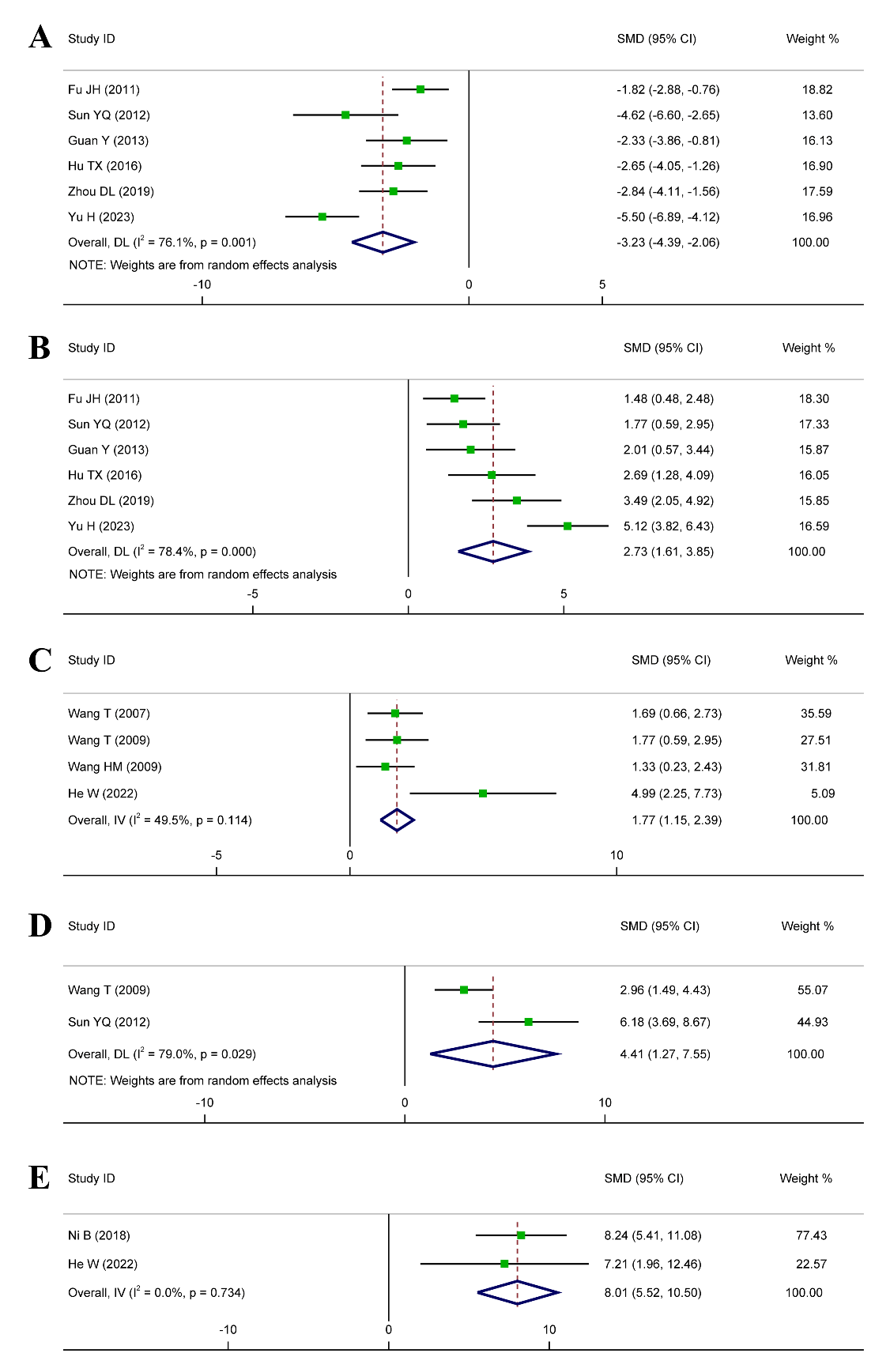
**

**Figure S2.** Forest plot presenting SMD and 95%CI for the effects of HSYA on oxidative stress. (A) MDA level; (B) SOD level; (C) NO level; (D) eNOS activity; (E) Nrf2 mRNA level.


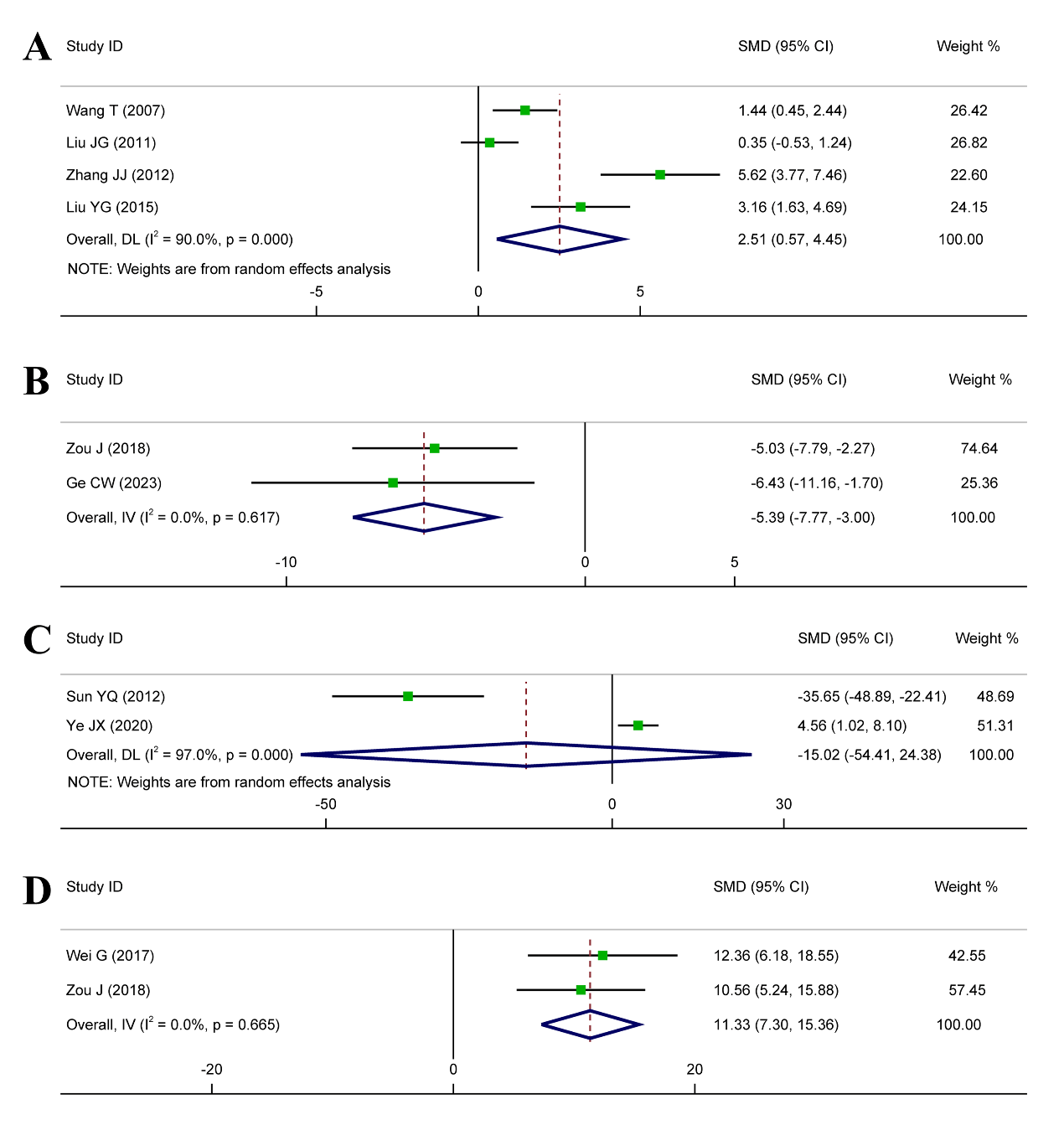


**Figure S3.** Forest plot presenting SMD and 95%CI for the effects of HSYA on 6-keto-PGF1α level (A), myocardium fibrosis (B), LC-3II/LC-3I ratio (C), and VEGFA mRNA level (D).


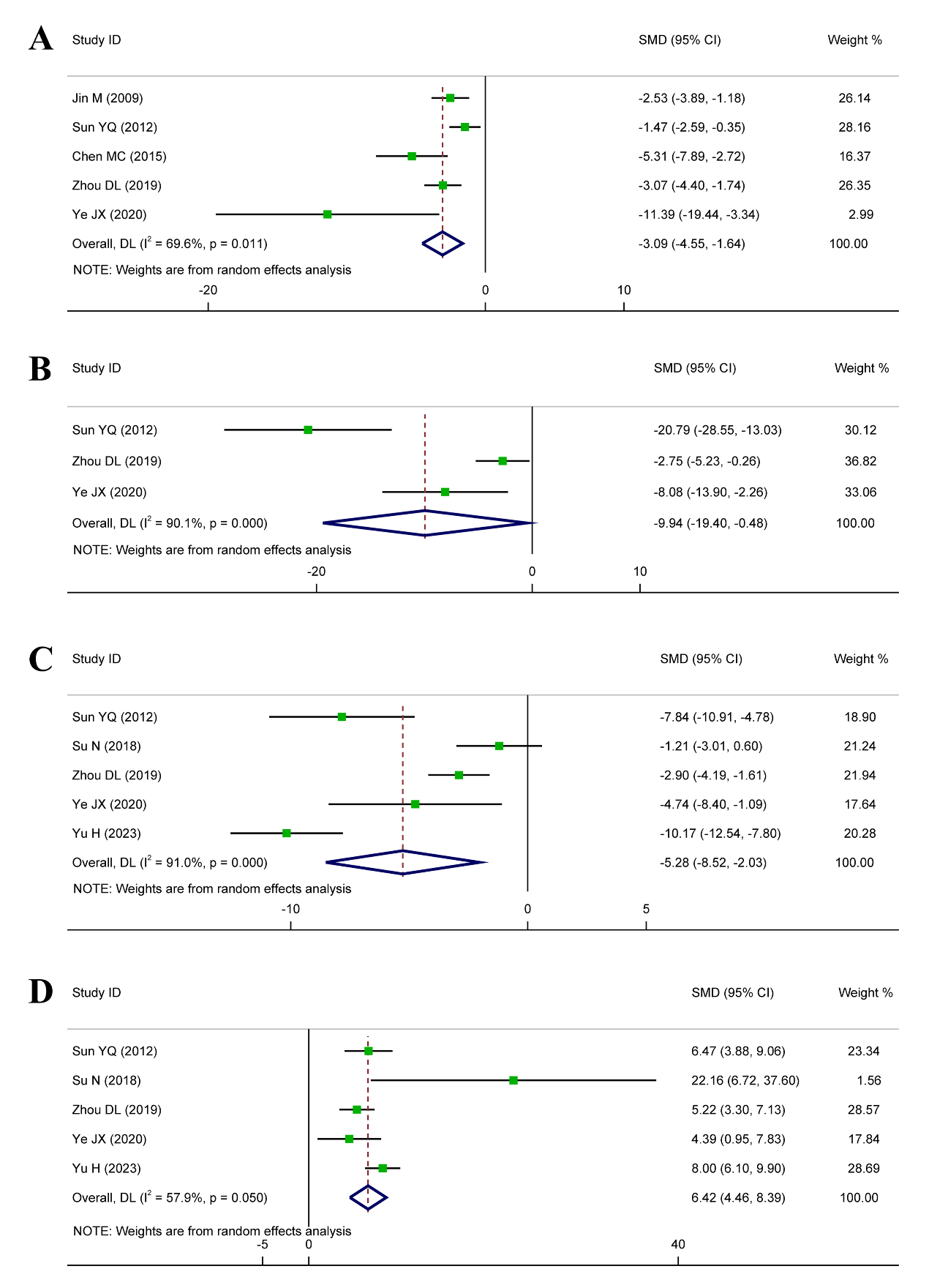


**Figure S4.** Forest plot presenting SMD and 95%CI for the effects of HSYA on apoptosis. (A) apoptosis index, (B) cleaved caspase-3 protein; (C) Bax protein; (D) Bcl-2 protein.


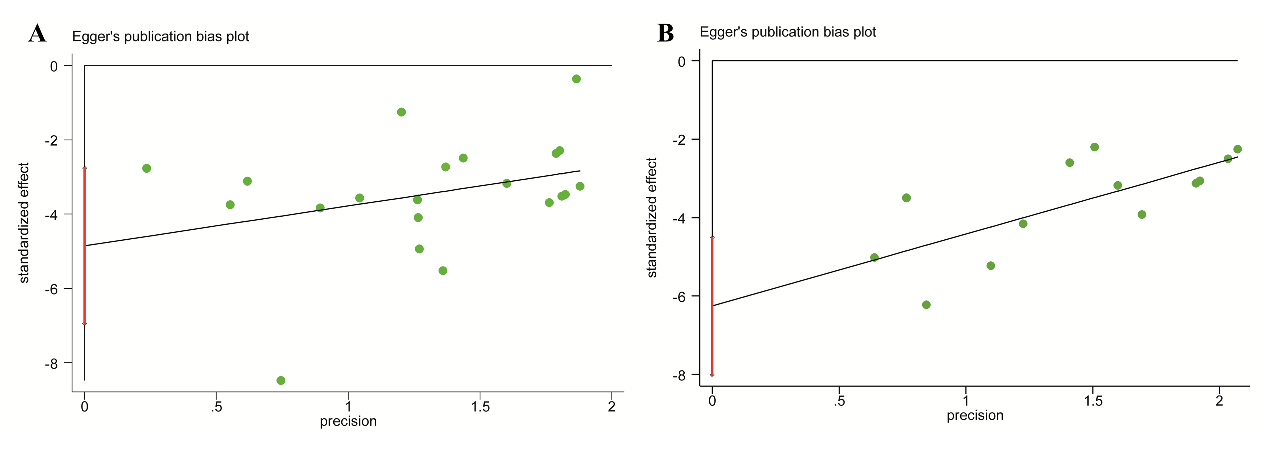


**Figure S5.** Egger’s test of MIS (A) and CK-MB (B).

**Table S1.** Detailed information of HSYA in each study.

| **Study ID** | **Source** | **Purity**  **(%)** | **Chemical analysis** |
| --- | --- | --- | --- |
| Wang T (2007) | Shandong Natural Medicine Engineering Technology Research Center, China | 98 | HPLC |
| Huang X (2009) | National Institute for the Control of Pharmaceutical and Biological Products, China | >99 | HPLC |
| Jin M (2009) | Department of Pharmacology, Beijing Anzhen Hospital, China | >92 | HPLC |
| Wang T (2009) | Shandong Luye Pharmaceutical, China | 98 | HPLC |
| Wang HM (2009) | Shandong Natural Medicine Engineering Technology Research Center, China | 98 | HPLC |
| Liu JG (2011) | Dalian Institute of Chemical Physics, Chinese Academy of Sciences, China | / | / |
| Fu JH (2011) | Taiyuan Huawei Pharmaceutical Co. Ltd, China | >98 | HPLC |
| Sun YQ (2012) | / | / | / |
| Zhang JJ (2012) | Sichuan Vikqi Biotechnology Co. Ltd, China | >98 | HPLC |
| Guan Y (2013) | National Institute for the Control of Pharmaceutical and Biological Products, China | ≥98 | HPLC |
| Dong WB (2014) | Zhejiang Yongning Pharmaceutical Co. Ltd, China | / | / |
| Xu AB (2014) | National Institute for the Control of Pharmaceutical and Biological Products, China | / | / |
| Zhou MX (2015) | Taiyuan Hua Wei Pharmaceutical Co. Ltd, China | >98 | HPLC |
| Chen MC (2015) | National Institute for the Control of Pharmaceutical and Biological Products, China | / | / |
| Hu TX (2015) | Chengdu Best Reagent Co. Ltd, China | / | / |
| Liu YG (2015) | Zhejiang Yongning Pharmaceutical Co. Ltd, China | / | / |
| Hu TX (2016) | National Institute for the Control of Pharmaceutical and Biological Products, China | >99 | HPLC |
| Wei G (2016) | Chengdu Best Reagent Co. Ltd, China | ≥96 | HPLC |
| Wei G (2017) | National Institute for the Control of Pharmaceutical and Biological Products, China | >98 | HPLC |
| Ni B (2018) | Shanghai Yuanye Biotechnology Co. Ltd, China | / | / |
| Zou J (2018) | Shanghai PureOne Biotechnology Co., Ltd, China | / | / |

**Tabel S1.** (Continued)

| **Study ID** | **Source** | **Purity**  **(%)** | **Chemical analysis** |
| --- | --- | --- | --- |
| Su N (2018) | Aladdin Reagent Co. Ltd, China | / | / |
| Zhou DL (2019) | Shanghai Yuanye Biotechnology Co., Ltd, China | ≥95 | HPLC |
| Ye JX (2020) | Shanghai Winherb Medical S&T Development, China | >98 | HPLC |
| Zhang Y (2020) | Dalian Meilun Biotechnology Co. Ltd, China | ≥98 | HPLC |
| He W (2022) | Aladdin Reagent Co. Ltd, USA | / | / |
| Ge CW (2023) | Tianjin Pacific Pharmaceutical Co. Ltd, China | >98 | HPLC |
| Yu H (2023) | Sichuan Vikqi Biotechnology Co. Ltd, China | / | / |
